# Flawed reports can harm: the case of supervised consumption services in Alberta

**DOI:** 10.17269/s41997-023-00825-x

**Published:** 2023-11-06

**Authors:** Ginetta Salvalaggio, Hannah Brooks, Vera Caine, Marilou Gagnon, Jenny Godley, Stan Houston, Mary Clare Kennedy, Brynn Kosteniuk, Jamie Livingston, Rebecca Saah, Kelsey Speed, Karen Urbanoski, Dan Werb, Elaine Hyshka

**Affiliations:** 1 Faculty of Medicine and Dentistry, University of Alberta, Edmonton, AB Canada; 2 School of Public Health, University of Alberta, Nashville, TN USA; 3 School of Nursing, University of Victoria, Victoria, BC Canada; 4 Department of Sociology, University of Calgary, Calgary, AB Canada; 5 British Columbia Centre On Substance Use, University of British Columbia - Okanagan School of Social Work, Vancouver, BC Canada; 6 School of Public Health, University of Alberta, Edmonton, AB Canada; 7 Department of Criminology, Saint Mary’s University, Halifax, NS Canada; 8 Department of Community Health Sciences, University of Calgary, Calgary, AB Canada; 9 School of Public Health, University of Alberta, Vancouver, BC Canada; 10 School of Public Health & Social Policy, University of Victoria, Victoria, BC Canada; 11 Centre On Drug Policy Evaluation, MAP Centre On Urban Health Solutions, St. Michael’s Hospital, Toronto, ON Canada; 12 Division of Infectious Disease & Global Public Health, University of California San Diego, La Jolla, CA USA

**Keywords:** Substance use, Addiction, Health policy, Public health, Consommation de substances, toxicomanie, politique de santé, santé publique

## Abstract

Supervised consumption services have been scaled up within Canada and internationally as an ethical imperative in the context of a public health emergency. A large body of peer-reviewed evidence demonstrates that these services prevent poisoning deaths, reduce infectious disease transmission risk behaviour, and facilitate clients’ connections to other health and social services. In 2019, the Alberta government commissioned a review of the socioeconomic impacts of seven supervised consumption services in the province. The report is formatted to appear as an objective, scientifically credible evaluation of these services; however, it is fundamentally methodologically flawed, with a high risk of biases that critically undermine its authors’ assessment of the scientific evidence. The report’s findings have been used to justify decisions that jeopardize the health and well-being of people who use drugs both in Canada and internationally. Governments must ensure that future assessments of supervised consumption services and other public health measures to address drug poisoning deaths are scientifically sound and methodologically rigorous. Health policy must be based on the best available evidence, protect the right of structurally vulnerable populations to access healthcare, and not be contingent on favourable public opinion or prevailing political ideology.

## Résumé

Les services de consommation supervisée ont été établis au Canada et à l’étranger en tant qu’impératif éthique dans le contexte d’une urgence de santé publique. Un grand nombre d’études rigoureuses démontrent que ces services préviennent les décès par empoisonnement, réduisent les comportements à risque de transmission de maladies infectieuses, et facilitent les liens avec d’autres services sociaux et de santé. En 2019, le gouvernement de l’Alberta a commandé un examen des impacts socioéconomiques de sept services de consommation supervisée dans la province. La présentation du rapport donne l’impression que l’évaluation de ces services est objective et scientifiquement crédible; cependant, il présente des faiblesses importantes au plan méthodologique, notamment en raison de la présence de biais qui compromet l’évaluation des preuves scientifiques. Ses conclusions ont été utilisées pour justifier des décisions qui mettent en péril la santé et le bien-être des personnes qui consomment des drogues, tant au Canada qu’à l’étranger. Les gouvernements doivent s’assurer que les futures évaluations des services de consommation supervisée et d’autres mesures de santé publique pour lutter contre les décès par empoisonnement dû aux drogues sont scientifiquement fondées. Les politiques en matière de santé doivent être basées sur les meilleures données disponibles, protéger les droits des populations structurellement vulnérables à accéder aux soins de santé, et ne pas dépendre de l’opinion publique ou d’une idéologie politique dominante.

## Introduction

People in Canada are dying from drug poisoning at an alarming rate; the immediate cause of the dramatic increase in deaths is the increasingly toxic unregulated drug supply (Public Health Agency of Canada, 2021). Harm reduction interventions such as supervised consumption services (SCS) are designed to reduce morbidity and mortality among people who use drugs (PWUD) (Health Canada, 2020). However, they remain controversial and are subject to greater scrutiny than other public health measures (Beattie, 2018). In 2019, the government of Alberta commissioned a pseudoscientific panel review to document the harms of SCS (Government of Alberta, 2020); the report has since been used to justify the closure of two Alberta SCS (Kalinowski, 2020; Mohatarem, 2021). An increase in drug poisoning events has been observed following both closures (Alberta Health, 2023). The report has been used in other settings to limit evidence-based responses to poisoning deaths (Tanenbaum, 2021). We call for the report to be retracted by its authors to reduce the risk of future deaths caused by misinformed policy.

## What is the evidence on supervised consumption services?

SCS are a core part of a public health response to an unprecedented poisoning epidemic that has resulted in 36,442 opioid toxicity deaths between January 2016 and December 2022, driven largely by the production and trafficking of novel illegally manufactured opioids (Public Health Agency of Canada, 2021). SCS provide monitored spaces where people can consume drugs without risk of criminal sanction, receive emergency health care if needed, and access sterile harm reduction supplies and health and social supports (Health Canada, 2020). The number of federally sanctioned SCS in Canada increased from two in 2016 to a peak of 42 in 2020 (Health Canada, 2020). Additionally, more than 40 overdose prevention sites—a low-threshold form of SCS meeting an immediate community need and requiring less pre-implementation consultation—have opened in Canada since 2016. Collectively, these services have prevented and managed thousands of drug poisoning events and saved thousands of lives (Irvine et al., 2019).

SCS are designed to reduce health and social risks, including risks associated with using drugs alone amid a toxic drug supply crisis. They also provide social support for structurally vulnerable populations who experience barriers to accessing health care (Kennedy et al., 2017). A substantial body of peer-reviewed research demonstrates the positive impacts of SCS. A systematic review by Kennedy et al. (with findings later corroborated by Levengood et al.) synthesized 47 studies from Vancouver, Australia, Germany, Denmark, Spain, and the Netherlands (Kennedy et al., 2017; Levengood et al., 2021). Studies adopted a mix of prospective cohort, time series or pre/post ecological, cross-sectional, mathematical simulation, or series cross-sectional designs and the majority were assessed to have good methodological quality. The review found that SCS mitigate drug poisoning–related harm and unsafe drug use practices, facilitate uptake of substance use treatment and other health services, are associated with improvements in public order (e.g., reductions in publicly discarded syringes), do not increase drug-related crime, and are cost-effective. A subsequent modelling study estimated that British Columbia’s overdose prevention sites averted 230 deaths in a 20-month period (Irvine et al., 2019). A study from Calgary, Alberta, found significant health system cost-savings arising from decreases in opioid-related ambulance responses and emergency department visits following the implementation of an SCS (Khair et al., 2022).

Even with robust research showing that SCS have strong public health benefits and minimal evidence of harm, negative perceptions persist in some settings. Public and political support for SCS is inconsistent across Canada, and local opposition has led to delays and cancellations of planned facilities (Renic & MacLean, 2020; Simon, 2019). In some jurisdictions, opponents have complained of perceived increases in improperly discarded syringes and criminal activity in surrounding areas, despite a lack of evidence (Beattie, 2018). In Alberta, where eight sites were implemented in five municipalities between 2017 and 2019, some individuals and organizations representing residents, businesses, law enforcement, and other stakeholders voiced criticism about potential adverse community impacts (Campbell, 2019). Given the large body of scientific evidence that refutes these concerns, the extent to which complaints and negative perceptions of SCS are legitimate—and whether they are a direct outcome of SCS provision versus a reflection of broader socioeconomic disparities, misinformation, and prejudice—needs to be rigorously examined.

Resolving these public tensions requires an objective assessment of individuals’ concerns, thoughtful consideration of steps to mitigate any issues identified, and concerted efforts to design SCS in a way that maximizes overdose prevention potential and its positive impact on surrounding communities. In 2019, Alberta’s governing United Conservative Party committed to conducting an “impact review” of the province’s SCS as part of its election platform and appointed a panel to carry out this review after forming government (Government of Alberta, 2020). However, the panel ultimately tabled a report in March 2020 that—though formatted and presented as a scientific study—failed to meet basic methodological criteria for credible research and evaluation, and contained serious flaws that critically undermined its conclusions.

## Why is the Alberta Government’s SCS review report not scientifically sound?

The review panel’s evaluation mandate was to assess the “socio-economic impacts of existing and proposed SCS sites on their host communities” (p. iii) (Government of Alberta, 2020). The eight panel members engaged in public in-person consultations, collected online data from a convenience sample, and examined SCS-related data and documents. Although touted as a rigorous review of SCS across the province, the panel’s 2020 report fits the following criteria associated with pseudoscience: (a) outcome reporting bias; (b) measurement bias; (c) confirmation bias; and (d) lack of independent peer review (Jakovljević & Ostojić, 2016).

*Outcome reporting bias* refers to bias stemming from selective data collection procedures and unbalanced reporting of results without adequate consideration of data refuting the findings. The Alberta government’s review was set up to have a high risk of outcome reporting bias given its stated aim “to minimize the adverse social and economic impacts of existing SCS sites on local neighbourhoods” (p. 2). Moreover, “the merits of SCS as a harm reduction tool” (p. 2) were outside the review’s scope (Government of Alberta, 2020). The review therefore pre-supposed that there were adverse impacts of SCS sites and explicitly excluded any positive health and social impacts of SCS from its assessment. The report also does not present a balanced account of the information collected throughout the review, emphasizing anecdotes that support the government’s ideological position on SCS and skimming over or excluding evidence that counters the government’s views. For example, the report references healthcare professionals who perceive SCS as “unethical” (p. 24), yet discredits other healthcare professionals for (correctly) citing evidence of minimal risk of injury from discarded needles (p. 5) (Government of Alberta, 2020). It does not include healthcare professional comments in support of SCS, as described by news media attending the panel’s town halls (Kindleman, 2019; Mendonsa, 2019). The report also relies on survey data in which one smaller city is disproportionately represented to support specific town hall narratives of perceived increases in crime and social disorder (p. 14); in contrast, survey respondents from larger centres report a net neutral impact from SCS on such metrics as perceived safety, public consumption, and discarded needles (pp. 51–54) (Government of Alberta, 2020). Unlike survey responses presented in the report’s appendices (pp. 51–188), transcripts of town hall events are not publicly available, leaving readers unable to confirm the trustworthiness of the review panel’s supplied interpretation of qualitative data. Thus, the reported outcomes and subsequent conclusions appear fundamentally skewed.

*Measurement bias* involves the systematically imprecise measurement of observed phenomena and can occur with both over-reliance on anecdotal evidence and faulty assumptions about the intervention under study. Detracting from the credibility of the commissioned review is a deliberate mischaracterization of SCS. For example, the report states that “the primary mandate of the SCS—preventing poisoning deaths—does not seem to apply to amphetamine use” and describes the use of SCS by people who use methamphetamine in negative terms (p. 14) (Government of Alberta, 2020), when, in fact, SCS are designed to minimize harms associated with any unregulated drug use (Alberta Health, 2020; Kennedy et al., 2017). The report also downplays oxygen as a vital intervention independent of naloxone co-administration. It suggests that non-naloxone emergency care does not constitute a poisoning response, excluding non-naloxone responses from its assessment of SCS-provided overdose interventions (p. 18) (Government of Alberta, 2020). This demonstrates a lack of understanding of the continuum of poisoning presentations and related care (Kerr et al., 2006), and is a dangerous assumption in an era of increasing non-opioid (e.g., benzodiazepine) contamination within Alberta’s drug supply. Finally, the report relies heavily on community anecdotes of SCS leading to “strange, aggressive, or bizarre behaviour” (p. 13) and “the appearance and especially the location of tent cities” (p. 6). The supplied anecdotes misattribute SCS as the cause of perceived social disorder despite the complex and intersecting circumstances faced by SCS clients such as homelessness, poverty, and other markers of structural vulnerability (Government of Alberta, 2020). While SCS can refer clients to external health and social services (e.g., housing, income support) (Kennedy et al., 2017), these are broader issues that SCS are not designed to address on their own. These statements demonstrate a poor understanding of SCS by the review panel itself, bringing the accuracy of the report’s supplied data and conclusions into question.

*Confirmation bias* is the over-weighting of hypothesis-supporting evidence and under-weighting of hypothesis-refuting evidence. Failure to situate results within existing literature that contradicts a study’s claims increases the risk of confirmation bias when drawing conclusions, and undermines the development of evidence-based recommendations (Jakovljević & Ostojić, 2016). The commissioned SCS report relies primarily on the selective data collected during the review and fails to draw on any prior Canadian or international literature, nor does it discuss whether its results support or refute findings from previous research or evaluations. While the review may be the first to find significant negative impacts of SCS, it is difficult to discern the validity of these observations in the absence of sound study methodology, and thus, it is impossible to situate the report within existing literature (Livingston, 2021). There is a high risk of confirmation bias given the discrepancy between the SCS report’s findings and the findings of other peer-reviewed evidence syntheses.

*Lack of peer review:* Finally, an absence of external, independent peer review calls into question the credibility of the report’s findings, as peer review is designed to detect and minimize errors and biases in methods and reasoning (Cowell, 2014). In a subsequent peer-reviewed critique of the report, Livingston (2021) identified many methodological flaws, including non-representative convenience sampling, inappropriate definitions and measurements, absence of inferential analyses of data, and misattribution of causality between SCS provision and socioeconomic outcomes (Livingston, 2021). While Livingston discussed these limitations in reference to crime data, they hold true for the other health and social data presented in the report, such as adverse medical events (p. 18) and addiction treatment access (p. 6) (Government of Alberta, 2020).

## What are the potential and real implications of this pseudoscientific report?

The Alberta government SCS review report has already been used to counter efforts to increase the availability of life-saving services for PWUD in Canada and internationally, and it may continue to reinforce harm. Following the March 2020 release of the report, the cancellation of all plans for proposed new SCS across Alberta was publicly announced. In August 2020, government funding was retracted from North America’s busiest SCS in Lethbridge, forcing it to close its doors (Kalinowski, 2020). The closure of the Boyle Street SCS in Edmonton followed in April 2021 (Mohatarem, 2021). Although peer-reviewed studies evaluating these site closures have not yet been published, the closures are temporally associated with impacts one would expect based on existing peer-reviewed literature; in Edmonton, for example, poisoning deaths increased from 47.4/100,000 person years/month in February–April 2021 (preceding the SCS closure) to 65.8/100,000 person years/month in May–July 2021 (following the SCS closure), a temporal trend that was not observed in Alberta’s other municipalities (Alberta Health, 2023). EMS responses and acute care utilization for poisoning events demonstrate similar temporally associated increases (Alberta Health, 2023). Instead of poisonings being managed in the community in a supervised, timely fashion to avoid poor outcomes, a health system witnessing unprecedented strain from the COVID-19 pandemic was handed the additional task of managing these avoidable acute care presentations.

Outside of Canada, the report has been cited in civil litigation concerning Safehouse, a proposed overdose prevention site in Philadelphia. The report was cited by the United States federal government as a lone piece of evidence demonstrating that SCS can have negative impacts on their surrounding communities (Safehouse, n.d.). In January 2021, the Third Circuit Court of Appeals ruled against motions to allow Safehouse to operate legally (Tanenbaum, 2021).

A similar, though failed, strategy was used against SCS in 2008 when the Government of Canada used opinion statements by federally funded law enforcement agencies to propose ending the legal exemption of Canada’s first SCS, Insite (Hyshka et al., 2013). Their decision resulted in a legal challenge, which culminated in the Supreme Court of Canada ruling that closing Insite would be a violation of the Charter of Rights and Freedoms for SCS clients (Hyshka et al., 2013). This earlier example highlights the real potential for reports unsubstantiated by sound research to undermine harm reduction service operations well beyond provincial jurisdiction.

## What can be done to mitigate the dangerous repercussions of this report?

It is critical that the report be retracted by the Alberta government, and that any of its scientifically trained authors distance themselves immediately from its contents. There is no room for a report with a high risk of multiple biases in making decisions affecting the health and well-being of structurally vulnerable populations and their communities. Rather, there is a need for a peer-reviewed, methodologically sound, and ethically principled evaluation of SCS in Alberta that accounts for both health and social benefits and potential harms and outlines strategies for balancing participant and community health and safety as needed. SCS, like other population health interventions, should continue to be scientifically evaluated to ensure that they are accomplishing their objectives and not causing unintended harm. However, assessments of SCS must use robust methodologies and fair evaluation criteria that prioritize the health of the target population, their families, and communities over public perceptions.

The continued existence of this pseudoscientific government report risks legitimizing similar approaches to future evidence generation and policy formation. In early 2022, the Alberta government proceeded with methodologically flawed evidence review activities concerning another harm reduction topic—safer supply—generating a June 2022 report intended to guide future government action (*Safe Supply Committee*, n.d.). Multiple independent academics and PWUD have publicly flagged the safer supply review process as pseudoscientific and potentially harmful (BCCSU, 2022). Indeed, rather than relying on peer-reviewed evidence, the Alberta government policy direction has shifted to align itself with the findings of its commissioned reports, while drug poisoning harms continue unabated; 803 more Albertans have died from drug poisoning in the first 5 months of 2023, which is potentially vying for status as the deadliest year on record (Alberta Health, 2023). Future topic reviews by legislators—especially for polarizing issues—should be conducted transparently with the support of independent experts in evaluation and review methods. All conflicts of interest should be declared. If the aim is to produce de novo evidence syntheses to guide decision-making, proposed review methodologies and methods and emerging reports must consider the context of the existing scientific literature, and be subject to external peer review.

## Conclusion

Despite peer-reviewed studies demonstrating that supervised consumption services prevent poisoning deaths and reduce other harms for PWUD, SCS continue to face opposition. The Alberta government’s pseudoscientific report—although presented as credible findings—is methodologically flawed with a high risk of multiple forms of bias. Drug policy should be based on the best available evidence, aim to protect structurally vulnerable populations’ access to healthcare, and not be contingent on prevailing ideology. There is an urgent need to shift away from commissioning reports that at their core seek to create moral panic. The Alberta government’s SCS review report could continue to be used to justify decisions that jeopardize the health and well-being of PWUD for years to come and erode trust in public health policies and practices.

## Data Availability

Not applicable.
